# Seed Morphology in Species from the *Silene mollissima* Aggregate (Caryophyllaceae) by Comparison with Geometric Models

**DOI:** 10.3390/plants11070901

**Published:** 2022-03-28

**Authors:** José Javier Martín-Gómez, Marco Porceddu, Gianluigi Bacchetta, Emilio Cervantes

**Affiliations:** 1 Instituto de Recursos Naturales y Agrobiología del Consejo Superior de Investigaciones Científicas (IRNASA-CSIC), Cordel de Merinas 40, E-37008 Salamanca, Spain; jjavier.martin@irnasa.csic.es (J.J.M.-G.); emilio.cervantes@irnasa.csic.es (E.C.); 2 Sardinian Germplasm Bank (BG-SAR), Hortus Botanicus Karalitanus (HBK), University of Cagliari (UNICA), Viale Sant’Ignazio da Laconi 9-11, 09123 Cagliari, Italy; bacchet@unica.it; 3 Centre for the Conservation of Biodiversity (CCB), Life and Environmental Sciences Department, University of Cagliari (UNICA), Viale Sant’Ignazio da Laconi 11-13, 09123 Cagliari, Italy

**Keywords:** biogeography, cardioid, islands, geometric models, Mediterranean flora, morphology, *Silene*, super-ellipse

## Abstract

The description of seed shape by comparison with geometric models allows shape quantification, providing the means for an accurate comparison between different species or populations. Geometric models described for the lateral and dorsal views of the seeds of *Silene* species are applied to the quantification of the shape in the seeds belonging to twenty populations of the eleven taxa of *S. mollissima* aggregate. Cardioid models LM1, LM5 and LM6 adjust differentially to the lateral views of the seeds, while models DM1, DM5 and DM6 are applied to the dorsal views of the seeds. Quantification of the lateral view of seeds with LM5 results in two groups of species of different geographic origin. The seeds more resembling DM5 include *S. andryalifolia*, *S. badaroi*, *S. gazulensis*, *S. hifacensis* and *S. tomentosa*, i.e., the taxa with a continental distribution from southern Spain to northern Italy; in contrast, the group of seeds with lower similarity to DM5 includes those from species in northern Africa and the Mediterranean Tyrrhenian islands: *S. auricolifolia*, *S. hicesiae*, *S. ichnusae*, *S. mollissima*, *S. oenotriae* and *S. velutina*. The description of the seed shape based on geometric models contributes to investigating the relationships between related species and constitutes a promising technique for taxonomy.

## 1. Introduction

The genus *Silene* L., with more than 700 species, includes an important proportion of the diversity found in the family Caryophyllaceae Juss. The taxonomy of this genus was traditionally based on morphological criteria, and more recently has received the support of intensive DNA sequence analysis [1]. The genus was divided in three subgenera: *Lychnis*, *Silene* and *Behenantha*. The subg. *Silene* contains 11 sections, including Sect. *Siphonomorpha* Otth, a large group comprising about 150 species of perennial diploids [2], including both the *S. italica* and *S. mollissima* aggregates [3,4,5].

The *S. mollissima* aggregate contains 11 species found in different biogeographic provinces along the western Mediterranean Basin [6,7]. Among them, five species are endemic to the Tyrrhenian islands macro-hotspot (sensu Cañadas et al. 2014 [8]); these are: *Silene badaroi* Breistr. (=*S. tyrrhenia* Jeanmonod & Bocquet), *S. ichnusae* Brullo, De Marco and De Marco f., *S. hicesiae* Brullo & Signor., *S. oenotriae* Brullo and *S. velutina* Pourr. ex Loisel. *Silene hifacensis* Rouy ex Willk. and *S. mollissima* (L.) Pers. are Ibero-Levantine endemic, and the remaining four species of this aggregate are endemic to the Baetic-Riffan macro-hotspot: *S. andryalifolia* Pomel, *S. auriculifolia* Pomel, *S. gazulensis* A.Galán, J.E.Cortés, Vicente Orell. & Mor. Alonso and *S. tomentosa* Otth. These species are currently identified by the morphological characters of their epigean vegetative parts (leaves, hairs), as well as their inflorescence, flowers and capsules [4,9].

The 11 species of the *S. mollissima* aggregate that are the subject of this work constitute a paradigm for the study of plant diversity in the Mediterranean area, a region characterized by high rates of endemism due to isolation, either in islands, through mountain ranges or due to geographic micro-environments [10]. Thus, *S. tomentosa* is endemic to Gibraltar, a small area with very specific characteristics [11], while *S. gazulensis* is endemic to the Sierra de los Gazules in the surroundings of Gibraltar [12]. *S. andryalifolia*, related to these species, has a broader distribution. The two Ibero-Levantine taxa, *S. hifacensis* and *S. mollissima,* are not sympatric. *S. hifacensis* only occurs on Ibiza and Alicante, while *S. mollissima* occurs on the Gymnesian islands of the Balearic Archipelago (Mallorca, Menorca and Cabrera) but not on the Pityusic islands (Ibiza or Formentera). Five of these species (*S. badaroi*, *S. ichnusae*, *S. hicesiae*, *S. oenotriae* and *S. velutina*) have a distribution centred in the Tyrrhenian area, with *S. badaroi* broadly distributed in the Provence and coast of north-western Italy; *S. velutina* is endemic to Sardinia and Corsica, and *S. ichnusae* is limited to north-western Sardinia while *S. hicesiae* and *S. oenotriae* occur in the southern Tyrrhenian sea. All these species are included in the section *Syphonomorpha* of subgen. *Silene* [1]. In this work, we have presented a morphological description of 20 populations from these species conserved in the Sardinian Germplasm Bank (BG-SAR; [13]) in order to find affinities between them as well as to investigate the variation in shape between species and populations.

Variations in the size and shape of the seeds represent an important source of information of high potential in taxonomy, particularly in *Silene* [14,15,16,17,18,19]. The application of image analysis techniques allows to obtain measurements related to the color, size and shape of seeds and to distinguish between different taxa or populations [20,21,22,23,24,25].

Based on previous studies on the seed morphology of the model plants *Arabidopsis thaliana* (L.) Heynh. [26,27], *Lotus japonicus* (Regel) K. Larsen and *Medicago truncatula* Gaertn. [28,29], seed shape descriptions based on the comparison with geometrical models have been applied to diverse plant genera and families [30,31,32], where the *J* index represents the percent of similarity between the geometric figure used as a model (cardioid) and the seed image. A striking similarity of the seed shape with the cardioid has been reported for the *Silene* species, in particular within *Silene* subg. *Behenantha*, with values of *J* index superior to 90 in a number of species (e.g., *S. noctiflora* L., *S. conica* L. and *S. latifolia* Poir.) [33]. In addition to the cardioid (Model 1, or LM1, where LM stands for lateral model), other models have already been described for the lateral views of *Silene* seeds [34,35]. LM2 is a flattened cardioid that demonstrated maximum similarity with the species *S. noctiflora* (*J* index 94.4) and *S. latifolia* (*J* index 93.0); LM3 was defined as an open cardioid curve having maximum similarity with *S. gallica* L. (*J* index 90.4) and LM4 was a flattened and elongated cardioid curve that indicated maximum similarity scores with *S. latifolia* (*J* index 92.5) and *S. diclinis* (*J* index 91.5) [33]. LM5 and LM6 are modified forms from model 3 with variations in the hilum region. Values of *J* index superior to 90 were obtained in *S. diversifolia* Otth and *S. tridentata* Ramond ex DC. [34]. Models LM7 and LM8 present other morphological peculiarities and provide *J* index values superior to 90 with *S. portensis* L. (both models), *S. nicaeensis* All., *S. littorea* Brot., *S. portensis* and *S. scabriflora* Brot. (model LM8) [34]. Models for the dorsal views of *Silene* seeds have also been described [35]. The *J* index values were more stable than the other typical morphological measures (i.e., area, length and width); hence, the association between the seed morphology and geometrical figures was stated as robust and could be used for classification purposes [33,34,35].

Concerning the seed morphology of the *S. mollissima* aggregate, morpho-colorimetric image analysis methods have been applied, revealing differences between the species [14,36]. By studying the seed morpho-colorimetric characteristics of *S. badaroi*, *S. ichnusae*, *S. hicesiae*, *S. oenotriae* and *S. velutina*, Murru et al. [36] reported that the current systematic treatment at the species level for these taxa is confirmed. However, the authors highlighted the need of further investigations regarding the taxonomic position of *S. hicesiae* in the whole aggregate as well as regarding the differentiation of *S. ichnusae* from *S. velutina* [36]. Nevertheless, the results from automated image analysis reported so far do not discriminate between the differences in size and shape, and the differences resulting from the analysis may be due to a mixture of shape, size or even color characteristics, while we are interested by the identification of differences in seed shape. The seeds of a given species or population can have a conserved shape that corresponds to a geometric model. This is detected by values of *J* index superior to 90 and has been reported in populations of diverse species of *Silene* [33,34,35].

Previous studies on the seeds’ characteristics in the taxa of a specific group membership proved that this type of analysis can be a useful tool for discriminating the species [23,24,29,35,36] or also in separating the taxa in different groups, such as, for example, the coastal species from the mountain ones [25]. These analyses are non-destructive, repeatable and, by incorporating the seed trait information with the leaves, hairs, flowers, fruit analysis or other morphological characteristics of a plant, may provide an important contribution for taxonomic studies.

Our objectives in this work are the following: (1) to describe the geometric models that define the seed shape in the species of *Silene mollissima* aggregate, and (2) to identify differences between the species in the shape of their seeds. We hypothesize that the new information provided by the geometric models analysis of seed shape may confer new knowledge useful to understand the relationships among species and to provide a new basis for the next taxonomic investigations regarding *Silene* taxa.

## 2. Results

### 2.1. General Morphology

#### 2.1.1. Lateral Views of Seeds

Table 1 presents a summary of the mean values and standard deviations for area (A), perimeter (P), length (L), width (W), aspect ratio (AR), circularity (C) and roundness (R) corresponding to the lateral views of the seeds in the 11 species of S. mollissima aggregate. Differences between the species were found in the size and shape for all the measurements. The mean area values were between 0.86 (*S. badaroi*) and 1.50 mm^2^ (*S. andryalifolia*). Three groups of different sizes were defined. The largest seeds corresponded to *S andryalifolia*, *S. auricolifolia* and *S. velutina*, the intermediate seeds to *S. hifacensis*, *S. ichnusae*, *S. mollissima*, *S. oenotriae* and *S. tomentosa* and the smallest seeds to *S. badaroi*, *S. gazulensis* and *S. hicesiae*.

The aspect ratio was between 1.15 in *S. auricolifolia* and 1.33 in *S. velutina*; three groups were defined corresponding to the rounded seeds with the smallest values of aspect ratio (*S. auricolifolia*), seeds with intermediate values (*S. andryalifolia*, *S. badaroi*, *S. gazulensis*, *S. hicesiae*, *S. hifacensis*, *S. ichnusae*, *S. mollissima* and *S. tomentosa*) and elongated seeds (*S. hicesiae*, *S. oenotriae* and *S. velutina*). The coefficient of variation had higher values in the measurements of size (A, P, L and W) than in the shape measurements (AR, C and R).

Different populations were analyzed in four species: *S. badaroi*, *S. hicesiae*, *S. hifacensis* and *S. velutina*. In the species with two populations analyzed (*S. hicesiae* and *S. velutina*), there was one population with larger seeds and the other with smaller seeds (not shown). There was no difference in aspect ratio, circularity or roundness in any of these two cases. There were also differences in seed size among the populations of *S. badaroi* (three populations) and *S. hifacensis* (six populations) (not shown). Differences in circularity were found between the populations of *S. badaroi* (not shown). In *S. hifacensis*, there were also differences in size, aspect ratio, circularity and roundness between the six populations studied (not shown).

#### 2.1.2. Dorsal View of Seeds

Table 2 presents a summary of the mean values and standard deviations for A, P, L, W, AR, C and R corresponding to the dorsal views of the seeds in the 11 species of *S. mollissima* aggregate. Differences between the species were found in size and shape for all the measurements. The mean area values were between 0.71 (*S. hicesiae*) and 1.51 mm^2^ (*S. auricolifolia*). Five groups of different sizes were defined. The largest seeds corresponded to *S. auricolifolia*, followed by the seeds of *S. andryalifolia*. The seeds of *S. oenotriae* and *S. velutina* were of intermediate size. *S. hifacensis*, *S. ichnusae*, *S. mollissima* and *S. tomentosa* were of small–intermediate size. The smallest seeds corresponded to *S. badaroi*, *S. gazulensis* and *S. hicesiae*.

Similar to the lateral view, the aspect ratio was between 1.08 in *S. auricolifolia* and 1.78 in *S. velutina*. Four groups were defined by the seeds with large (*S. gazulensis*, *S. hicesiae*, *S. hifacensis*, *S. mollissima* and *S. velutina*), intermediate (*S. badaroi* and *S. ichnusae*), intermediate–small (*S. andryalifolia*, *S. oenotriae* and *S. tomentosa*) and smallest values of aspect ratio (*S. auricolifolia*). The coefficient of variation had higher values in the measurements of size (A, P, L and W) than in the shape measurements (AR, C and R).

In the species with two populations analyzed (*S. hicesiae* and *S. velutina*), there was no difference in size, aspect ratio, circularity or roundness between the populations (not shown). In contrast, there was a difference in the size and aspect ratio among the populations of *S. badaroi* (three populations) and *S. hifacensis* (six populations) (not shown).

### 2.2. Comparison of the Average Silhouette of Seeds with the Geometric Models

#### 2.2.1. Lateral View of Seeds

**Figure 1:**
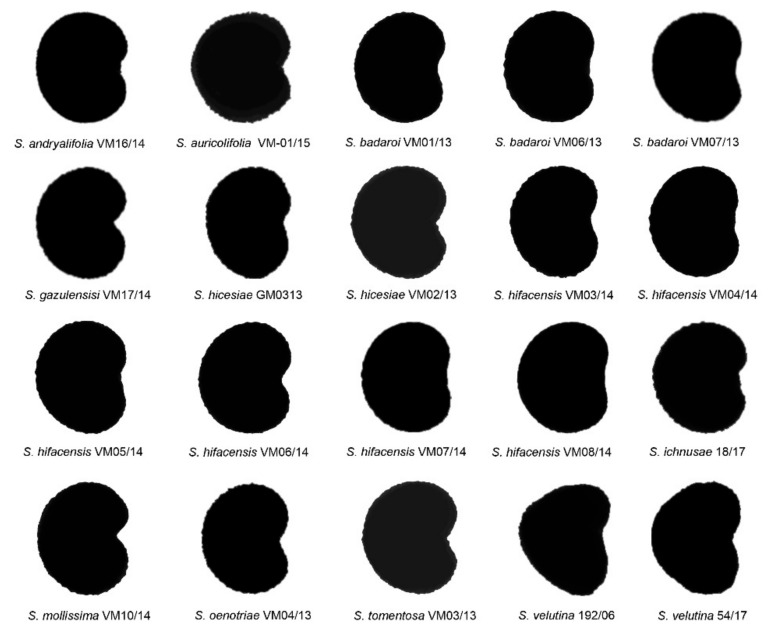
Average silhouettes obtained for the lateral views of 20 populations of 11 species in the *Silene mollissima* aggregate.

The average silhouettes obtained for the lateral view of the seeds in all 20 populations belonging to 11 species are shown in Figure 1. They were compared with the models LM1 to LM8 described in Refs. [33,34].

The average silhouette of each population was compared with the eight geometric models. The results of the comparison are given in Table 3. The values of *J* index (percent similarity to the models) superior to 90 were obtained in all the species tested. Maximum values were obtained with model LM1 for *S. andryalifolia*, *S. auricolifolia* and *S. ichnusae*, with model LM4 for *S. badaroi*, *S. hicesiae*, *S. hifacensis* and *S. oenotriae* and with model LM5 for *S. gazulensis, S. mollissima* and *S. tomentosa*. *S. velutina* showed the best score with model LM8. The *J* index was calculated with models LM1, LM4 and LM5 for all the species.

#### 2.2.2. Dorsal View of Seeds

**Figure 2:**
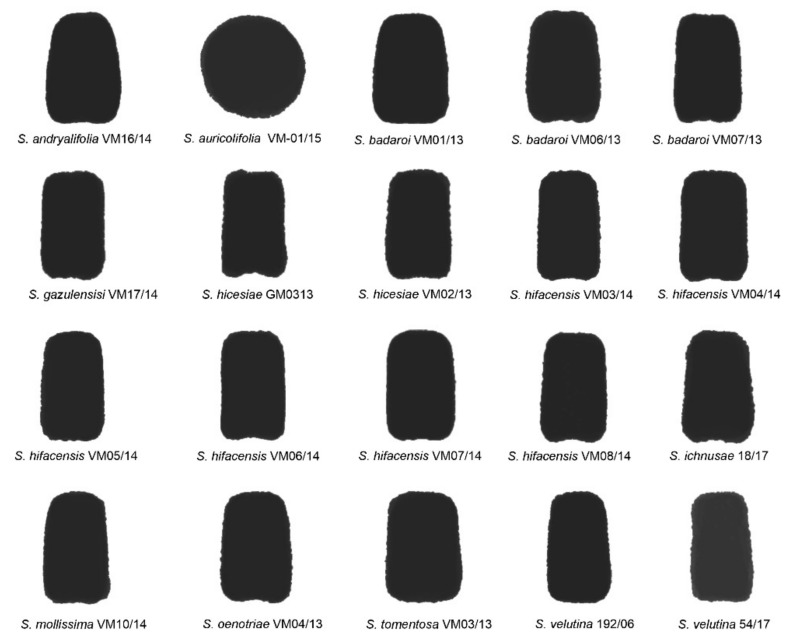
Average silhouettes obtained for the dorsal views of 20 populations of 11 species in the *Silene mollissima* aggregate.

The average silhouettes obtained for the dorsal view of the seeds in all 20 populations belonging to 11 species are shown in Figure 2. They were compared with models DM1, DM5 and DM6 [35].

The results of the comparison of the average silhouette of each species in the dorsal views of the seeds with the three geometric models tested are given in Table 4. Values superior to 90 were obtained for all the species except for *S. auricolifolia*, whose seeds are round in their lateral view. Maximum values were obtained with model DM5 for *S. andryalifolia*, *S. badaroi*, *S. oenotriae* and *S. tomentosa*. With model DM6, the highest values were obtained for *S. gazulensis*, *S. hicesiae*, *S. hifacensis*, *S. ichnusae*, *S. mollissima* and *S. velutina*. The *J* index values were tested with models DM1, DM5 and DM6 for the seeds of all the species.

### 2.3. Shape Quantification with Models: The Mean J Index in the Seeds of Each Species

#### 2.3.1. Shape Quantification in the Lateral Views with Models LM1, LM4 and LM5

The percent similarity (*J* index) of the seeds with the models giving the highest values in the comparison with the average silhouettes (LM1, LM4 and LM5) was obtained for all the species (Table 5).

**Figure A1:**
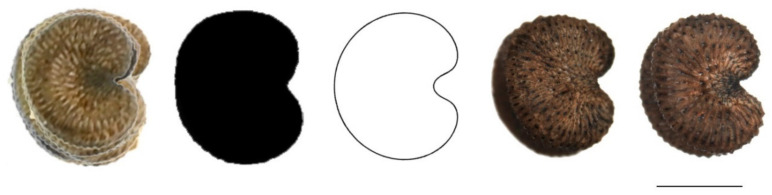
Superimposed images of the lateral view of *Silene andryalifolia* seeds, the corresponding average silhouette, model LM5 and two seeds of this species. Bar represents 1 mm.

**Figure A2:**
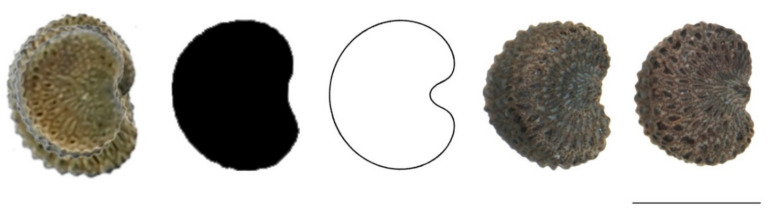
Superimposed images of the lateral view of *Silene badaroi* seeds, the corresponding average silhouette, model LM5 and two seeds of this species. Bar represents 1 mm.

**Figure A3:**
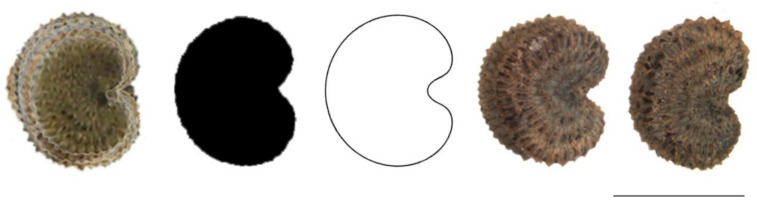
Superimposed images of the lateral view of *Silene gazulensis* seeds, the corresponding average silhouette, model LM5 and two seeds of this species. Bar represents 1 mm.

**Figure A4:**
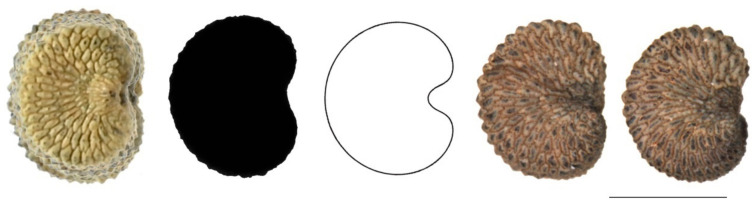
Superimposed images of the lateral view of *Silene hifacensis* seeds, the corresponding average silhouette, model LM5 and two seeds of this species. Bar represents 1 mm.

**Figure A5:**
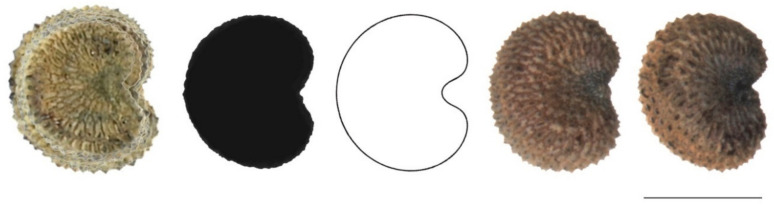
Superimposed images of the lateral view of *Silene tomentosa* seeds, the corresponding average silhouette, model LM5 and two seeds of this species. Bar represents 1 mm.

**Figure A6:**
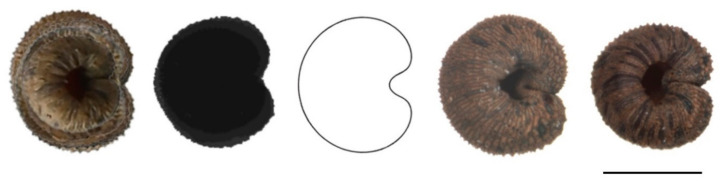
Superimposed images of the lateral view of *Silene auricolifolia* seeds, the corresponding average silhouette, model LM5 and two seeds of this species. Bar represents 1 mm.

**Figure A7:**
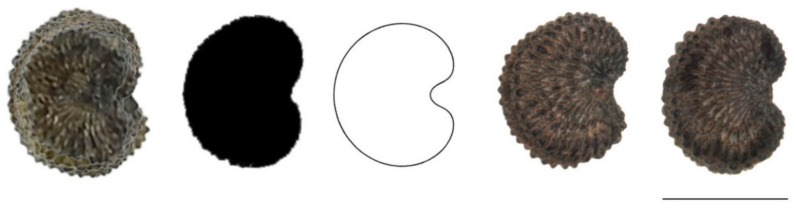
Superimposed images of the lateral view of *Silene hicesiae* seeds, the corresponding average silhouette, model LM5 and two seeds of this species. Bar represents 1 mm.

**Figure A8:**
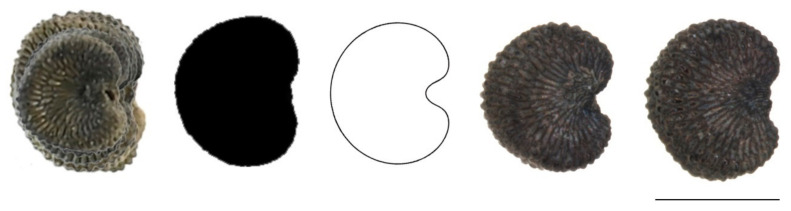
Superimposed images of the lateral view of *Silene ichnusae* seeds, the corresponding average silhouette, model LM5 and two seeds of this species. Bar represents 1 mm.

**Figure A9:**
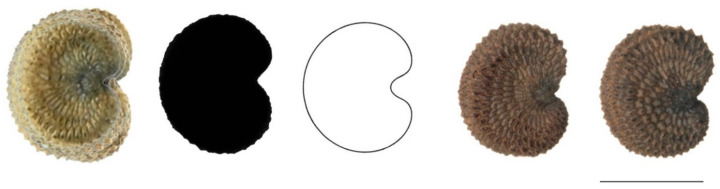
Superimposed images of the lateral view of *Silene mollissima* seeds, the corresponding average silhouette, model LM5 and two seeds of this species. Bar represents 1 mm.

**Figure A10:**
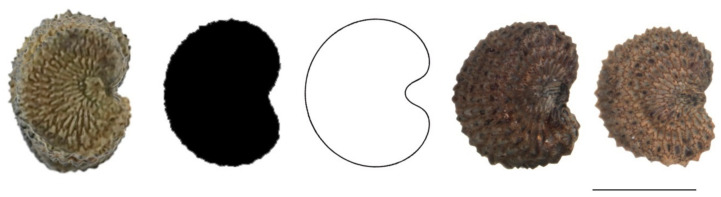
Superimposed images of the lateral view of *Silene oenotriae* seeds, the corresponding average silhouette, model LM5 and two seeds of this species. Bar represents 1 mm.

**Figure A11:**
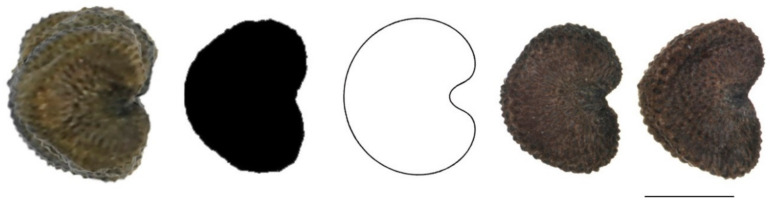
Superimposed images of the lateral view of *Silene velutina* seeds, the corresponding average silhouette, model LM5 and two seeds of this species. Bar represents 1 mm.

The analysis with each of the three models revealed a variable number of groups of low, intermediate and high *J* index values. Three species gave the lowest scores with model LM1 (*S. hicesiae*, *S. oenotriae* and *S. velutina*), and *S. auricolifolia* gave the highest values. Intermediate values of *J* index with LM1 were obtained in the remaining species. Three species gave the lowest scores with model LM4 (*S. auricolifolia, S. mollissima* and *S. velutina*), while the highest values were observed in *S. hifacensis* followed by *S. badaroi* and *S. tomentosa*; the remaining species had intermediate values. With model LM5, two groups were obtained of low (*S. auricolifolia*, *S. hicesiae*, *S. ichnusae*, *S. mollissima*, *S. oenotriae* and *S. velutina*) and high values of *J* index (*S. andryalifolia*, *S. badaroi*, *S. gazulensis*, *S. hifacensis* and *S. tomentosa*). Images of LM5 with *S. andryalifolia*, *S. badaroi*, *S. gazulensis*, *S. hifacensis* and *S. tomentosa* are shown in Figure A1, Figure A2, Figure A3, Figure A4 and Figure A5 of Appendix A, and images of LM5 with *S auricolifolia*, *S. hicesiae*, *S. ichnusae, S. mollissima*, *S. oenotriae* and *S. velutina* are shown in Appendix A Figure A6, Figure A7, Figure A8, Figure A9, Figure A10 and Figure A11, respectively.

The coefficient of variation had lower values in the measurements of *J* index than in the other shape measurements (AR, C and R; see Table 1).

#### 2.3.2. Shape Quantification in the Dorsal Views with Models DM1, DM5 and DM6

The percent similarity of the dorsal views of the seeds with models DM1, DM5 and DM6 (*J* index) was obtained for all the species (Table 6).

The analysis with model DM1 revealed four groups: the highest scores were obtained in *S. tomentosa*, lowest in *S. auricolifolia* followed by *S. velutina* and intermediate in the remaining species. Nevertheless, better scores were obtained with DM5 and DM6. The values with model DM5 defined four groups: one formed by *S. andryalifolia*, *S. badaroi* and *S. tomentosa* with the highest scores, the lowest scores in *S. auricolifolia* followed by *S. velutina* and intermediate in the remaining species. The DM6 demonstrated the highest values of the *J* index in *S. mollissima* followed by *S. hifacensis*, with the lowest scores in *S. auricolifolia* followed by *S. tomentosa* while the remaining species had intermediate values. The coefficient of variation had lower values in the measurements of *J* index than in the other shape measurements (AR, C and R; see Table 2).

## 3. Discussion

The description of the seed shape by comparison with geometric models allows the quantification of shape, an important step for phenotype characterization in the analysis of differences between varieties and species as well as for the study of the effect of environmental factors. The information reported in this study allows to increase the knowledge on the seed traits of species of *Silene* [15,16,18,33,34], with particular reference to the members of *Silene mollissima* aggregate [36]. The overall seed shape in the lateral views of the *Silene* species resembles a cardioid and figures related to it, while, in the dorsal views, it is more elongated, resulting in shapes related to modified ellipses and hyper-ellipses. The models used in this work for the lateral views of the seeds (LM1 to LM8) were described by Martín-Gómez et al. and Juan et al. [33,34], while the models DM1, DM5 and DM6, used in the description of the dorsal views of the seeds, were recently described by Rodriguez-Lorenzo et al. [35]. This is the fourth publication reporting seed shape quantification in species of *Silene* by this method and the first time that a set of lateral and dorsal models has been applied simultaneously to a set of geographically and phylogenetically related species.

The first conclusion of this work confirms the previous observations detected by using this type of analysis (e.g., [33,34,35]) and especially refers to the stability of shape. In summary, (I) the coefficient of variation is smaller in shape than in size measurements, and (II) the coefficient of variation is smaller in a measurement that describes the overall similarity of a seed image with a geometric figure (*J* index) than in general shape measurements (aspect ratio, circularity or roundness). In this work, differences in the size between populations were found in *S. badaroi*, *S. hicesiae*, *S. hifacensis* and *S. velutina*, while differences in the shape estimated as *J* index values with the different models were found only in the comparisons made with average silhouettes.

The differences in the size between the populations of the same species could not be correlated with known geographical factors. The populations having different seed size of species *S. badaroi*, *S. hicesiae*, *S. hifacensis* and *S. velutina* were located close to each other to attribute differences to climatic features, such as mean minimum temperatures or latitude. In the other cases reported, the differences in seed size in *S. dioica* were attributed to local, micro-environmental effects acting on a group of plants [37], similar to those described in *Arabidopsis helleri* (L.) O’Kane & Al-Shehbaz due to the presence of metals in the soil [38]. Differences in size between populations can also be attributed to the date of collection; for example, the seeds produced earlier in a plant are larger than seeds produced later [39].

A linear discriminant analysis (LDA) based on morpho-colorimetric measurements was applied to five Tyrrhenian species of the *S. mollissima* aggregate [36]. In this work, *S. badaroi* and *S. hicesiae* were separated from each other and from the other species analyzed (*S. ichnusae*, *S. oenotriae* and *S. velutina*). There are multiple factors influencing the result of LDA, while our analysis addresses more precisely the question of overall seed shape differences between species or populations independent of other considerations of size or color measurements.

The 11 species from the *S. mollissima* aggregate can be included in two groups for the lateral views and three for the dorsal views of seeds. Concerning the lateral views, the first group showing higher values of *J* index with model LM5 includes *S. andryalifolia, S. badaroi*, *S. gazulensis*, *S. hifacensis* and *S. tomentosa*; the second group, with lower values of *J* index with LM5, includes *S. auricolifolia, S. hicesiae*, *S. ichnusae*, *S. mollissima*, *S. oenotriae* and *S. velutina. S. gazulensis* is endemic in the Gibraltar area and its relatedness with *S. tomentosa* was already reported in the description of the former species [35].

In relation to the dorsal views, three groups resembling the results of the analysis of the aspect ratio were obtained. First, *S. auricolifolia*, with rounded seeds, forms an independent group. The second group contains all the species whose seeds have intermediate values of aspect ratio and whose dorsal views resemble model DM5 (*S. andryalifolia*, *S. badaroi*, *S. oenotriae* and *S. tomentosa*) and the third group, similar to species with higher values of aspect ratio, contains seeds resembling in their dorsal view the model DM6 (*S. gazulensis*, *S. hicesiae*, *S. hifacensis*, *S. ichnusae*, *S. mollissima* and *S. velutina*). According to their lateral and dorsal shape, and in relation to the other species studied by this method, the species of the *S. mollissima* aggregate resemble *S. conica* and *S. coutinhoi,* whose dorsal views adjust well to DM5 and DM6, respectively [35]. These two models have similar figures, with the only difference being that DM6 is narrower than DM5. The Iberian species *S. coutinhoi* is closely related to these species in the *Silene* sect. *Siphonomorpha* [40].

The analysis carried out in this work permitted to describe the seed shape of 20 populations of 11 taxa of *S. mollissima* aggregate as well as to detect two groups for the lateral views and three groups for the dorsal views of seeds of species of different geographic origin. The description of the seed shape by comparison with geometric models should be used as a complementary method in studies that take into consideration other morphological characteristics, such as the morpho-colorimetric quantitative and qualitative features of seeds, leaves, hairs, flowers, fruit or other characteristics of a plant, in order to ensure a more effective performance of classification among taxa. It is a low-cost and non-invasive approach, may contribute to the future studies on the other species of *Silene* and could be useful as the basis for a large-scale study.

## 4. Materials and Methods

### 4.1. Seeds of Silene Analyzed

**Figure 3:**
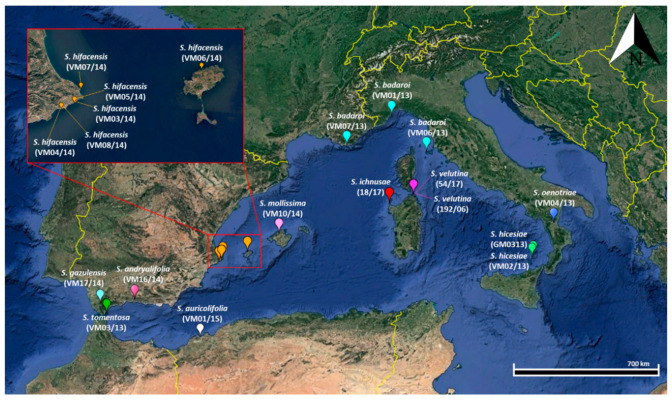
Map of the western Mediterranean biogeographic region showing the localities of origin of the seed populations used in this work.

Seeds of the 11 species of the *Silene mollissima* aggregate were obtained in the Sardinian Germplasm Bank (BG-SAR; [40]) and are described in Table 7. The localities of origin of the seed populations used in this work are shown in Figure 3. The accessions of each species were stored at −25 °C and preserved on the basis of established international protocols [41,42].

### 4.2. Seed Images

Photographs were taken with a Nikon Z6 camera with an objective AF-S Micro NIKKOR 60 mm f/2.8G ED. From an initial photograph made to the pool of seeds in each population and containing between 30 and 110 seeds, composed images containing 20–30 seeds regularly oriented per accession were prepared with Corel Photo Paint. In these images, the seeds were aligned to allow further analysis in ImageJ. The images are stored at: https://zenodo.org/record/6205212#.YhOpUujMKM8 (accessed on 21 February 2022).

### 4.3. General Morphological Description

Area (A), perimeter (P), length of the major axis (L), width (W), aspect ratio (AR is equal to L/W), circularity (C) and roundness (R) were obtained for the lateral views of seeds of each species from the initial photographs containing 16–110 seeds with ImageJ program [43]. The seeds were oriented with the micropyle to the right. A ruler was the reference for the conversion of pixel units to length or surface units (mm or mm^2^). The circularity index and roundness were calculated as described [44]. Circularity is the ratio (4π × A)/P2, while roundness is (4 × A)/πL2; in consequence, circularity decreases with irregularities of seed surface that increase the perimeter, but roundness is not affected.

### 4.4. Obtention of an Average Silhouette

The average silhouette is a representative image of seed shape for each group of seeds. A total of 20 seeds were used for each studied species. The silhouette was obtained in Corel Photo Paint by the protocol described [45] (a detailed video is available at Zenodo: https://zenodo.org/record/4478344#.YBPOguhKiM8, accessed on 2 September 2021). The layers containing the seeds are superimposed and the opacity is given a value of 20 in all layers. All the layers are combined, and the brightness is adjusted to a minimum value. From this image, we are interested in the inner region representing the area where most of the seeds coincide, which is the darkest area. To select it, we use the magic wand tool and, with tolerance equal to 10, this selection is copied and pasted as a new layer.

### 4.5. Geometric Models Used in the Comparisons

The models used in this work were: for the lateral views of seeds, the eight models already described for *Silene* seeds [33,34] that are stored in Zenodo https://zenodo.org/record/5535612#.YbyrC2jMKM8 (accessed on 21 February 2022).

Models DM1, DM5 and DM6 for the dorsal views of seed models are available at: https://zenodo.org/record/5997355#.Yg0zSN_MKM8 (accessed on 21 February 2022).

### 4.6. Comparison with Geometric Models: Calculation of the J Index

*J* index is defined by:

*J* index = (area S)/(area T) × 100

where S is the area shared between the seed image and the model and T, the total area occupied by both figures. *J* index ranges between 0 and 100, reaching maximum values when the geometric model and the seed image areas coincide. A high value of *J* index, i.e., high similarity with a given model, means a precise definition of seed shape for a particular species. A good adjustment to the model was considered when *J* index values were superior to 90 [33].

*J* index was calculated with the eight geometric models described for *Silene* on the average silhouettes of each species. Those models giving high values were applied to calculations of *J* index with the samples of 20–30 seeds.

**Figure 4:**
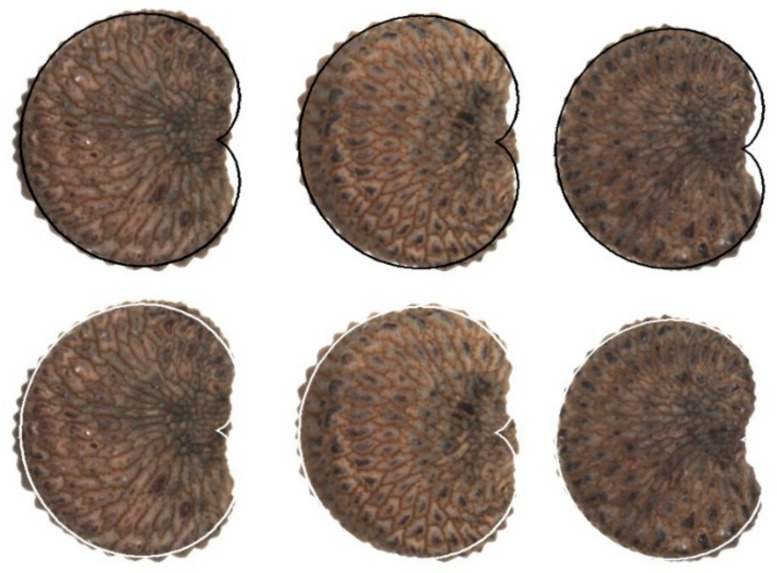
A sample of the composed images used for *J* index calculation. Seed images are combined with the geometric figure (model) in a different layer. In the upper part of this image, three seeds of *Silene mollissima* have the cardioid superimposed in black. They give the value of total area in ImageJ. The same seed images with model superimposed in white give the values of shared area between the seed and the model.

Area calculation was carried out by superimposing the model on each seed image, searching a maximum adjustment between the shapes of the seeds and the geometric model (Figure 4). Three files were kept for each composition: (1) a document (PSD format) with the seeds and the geometric figure adapted to each of them, in which it is possible to make corrections; (2) a file (JPG format) with the geometric models in black, which is useful to obtain total area (T) with the software ImageJ and (3) another file in JPG format with the geometric models in white, useful to obtain the values of area shared between the geometric figure and the seed image (S) in ImageJ (Figure 4). Image composition with seeds and models was done in Corel PHOTO-PAINT X7, and area quantification in ImageJ. Figure 4 illustrates examples of the adjustment between seed images and the geometric models with indication of the areas measured for the calculation of the *J* index.

### 4.7. Statistical Analysis

The raw data are available at: https://zenodo.org/record/6378816#.YjrjbOfMKM8 (accessed on 23 March 2022). The mean, minimum and maximum values and the standard deviation were obtained for all the measurements indicated (A, P, L, W, AR, C and R) as well as for *J* index with the different models. Statistics were analyzed on IBM SPSS statistics v28 (SPSS 2021) and R. software v. 4.1.2 [46]. As some of the populations did not follow a normal distribution, non-parametric tests were applied for the comparison of populations. Kruskal–Wallis test was completed followed by stepwise stepdown comparisons by the ad hoc procedure developed by Campbell and Skillings [47]. P values inferior to 0.05 were considered significant. The coefficient of variation was calculated as CVtrait = standard deviationtrait/meantrait × 100 [48].

## 5. Conclusions

The seed morphology in the 11 species of the *S. mollissima* aggregate has been analyzed based on the comparison with geometric models both in the lateral and dorsal views. In the lateral view, the seeds resemble a cardioid (LM1) or models derived from it (LM4 and LM5). The comparison with LM5 revealed two groups of high and low similarity with this model. The group of high similarity to LM5 contains the species from Andalousie and Levant in southern Spain and the region of the Mediterranean coast in France and northern Italy with *S. andryalifolia*, *S. badaroi*, *S. gazulensis*, *S. hifacensis* and *S. tomentosa.* The group of lower similarity to LM5 includes the species of the islands along the Mediterranean Sea and north Africa: *S. auricolifolia, S. hicesiae*, *S. ichnusae*, *S. mollissima*, *S. oenotriae* and *S. velutina*. *S. auricolifolia* constitutes a separate group due to roundness in both the lateral and dorsal views.

In comparison with the other species analyzed by geometric models, and in particular in their dorsal views, the seeds of the *S. mollissima* aggregate, excluding *S. auricolifolia*, resemble species *S. conica* and *S. coutinhoi*.

Differences between the populations of the same species were found for seed size, circularity and roundness but not in shape quantification by similarity with a geometric model (*J* index), thus indicating the stability of the shape measured by comparison with a geometric model in comparison with the other measurements.

## Figures and Tables

**Table 1:** Results of Kruskal–Wallis and post hoc tests for the size and shape measurements in the lateral views of seeds in species of the *Silene mollissima* aggregate. Mean values and coefficient of variation (given in parentheses) are indicated for area (A), perimeter (P), length (L), width (W), aspect ratio (AR), circularity (C) and roundness (R). Values marked with the same superscript letter in each column correspond to populations that do not differ significantly at *p* < 0.05. N indicates the number of seeds analyzed.

Species	N	A (mm^2^)	P (mm)	L (mm)	W (mm)	AR	C	R
*S. andryalifolia*	30	1.50 ^e^ (9.28)	4.98 ^f^ (5.02)	1.55 ^e^ (5.29)	1.23 ^d^ (5.22)	1.27 ^bcd^ (5.06)	0.76 ^d^ (3.60)	0.79 ^bcd^ (4.67)
*S. auricolifolia*	15	1.44 ^de^ (18.80)	4.89 ^f^ (10.98)	1.45 ^d^ (9.31)	1.26 ^d^ (9.87)	1.15 ^a^ (5.17)	0.75 ^cd^ (6.27)	0.87 ^e^ (4.97)
*S. badaroi*	130	0.86 ^a^ (16.03)	3.77 ^a^ (8.50)	1.17 ^a^ (8.34)	0.93 ^a^ (8.40)	1.25 ^b^ (4.88)	0.76 ^d^ (4.00)	0.80 ^cd^ (4.82)
*S. gazulensis*	48	0.90 ^a^ (11.74)	4.01 ^c^ (6.57)	1.22 ^b^ (6.63)	0.94 ^a^ (7.01)	1.28 ^cde^ (7.40)	0.69 ^a^ (4.24)	0.77 ^abc^ (7.08)
*S. hicesiae*	130	0.87 ^a^ (14.91)	3.90 ^b^ (7.45)	1.21 ^b^ (8.25)	0.92 ^a^ (8.50)	1.32 ^e^ (8.30)	0.72 ^b^ (4.12)	0.76 ^a^ (8.11)
*S. hifacensis*	282	1.19 ^bc^ (18.18)	4.49 ^d^ (10.37)	1.38 ^c^ (9.46)	1.09 ^b^ (9.20)	1.26 ^bc^ (4.11	0.74 ^c^ (5.99)	0.80 ^cd^ (4.05)
*S. ichnusae*	66	1.23 ^bc^ (17.21)	4.56 ^de^ (9.79)	1.41 ^cd^ (10.38)	1.11 ^b^ (8.74)	1.25 ^bc^ (7.97)	0.75 ^c^ (5.39)	0.79 ^cd^ (7.61)
*S. mollissima*	40	1.16 ^bc^ (13.57)	4.58 ^de^ (6.36)	1.36 ^c^ (6.78)	1.08 ^b^ (7.09)	1.26 ^bc^ (3.33)	0.69 ^a^ (3.71)	0.80 ^cd^ (3.35)
*S. oenotriae*	61	1.23 ^c^ (14.59)	4.80 ^f^ (7.23)	1.43 ^d^ (8.87)	1.09 ^b^ (9.72)	1.32 ^de^ (11.26)	0.68 ^a^ (5.50)	0.77 ^ab^ (10.73)
*S. tomentosa*	44	1.15 ^b^ (10.38)	4.62 ^e^ (6.28)	1.34 ^c^ (5.17)	1.09 ^b^ (6.38)	1.23 ^b^ (4.54)	0.68 ^a^ (4.87)	0.81 ^d^ (4.61)
*S. velutina*	135	1.38 ^d^ (15.01)	4.83 ^f^ (7.91)	1.53 ^d^ (8.43)	1.15 ^c^ (9.70)	1.33 ^e^ (9.82)	0.74 ^c^ (3.98)	0.76 ^a^ (9.80)

**Table 2:** Results of Kruskal–Wallis and post hoc tests for the size and shape measurements of the seeds in species of the *Silene mollissima* aggregate. Mean values and coefficient of variation (given in parentheses) are indicated for area (A), perimeter (P), length (L), width (W), aspect ratio (AR), circularity (C) and roundness (R). Values marked with the same superscript letter in each column correspond to populations that do not differ significantly at *p* < 0.05. N indicates the number of seeds analyzed.

Species	N	A (mm^2^)	P (mm)	L (mm)	W (mm)	AR	C	R
*S. andryalifolia*	24	1.33 ^d^ (8.90)	4.88 ^d^ (5.91)	1.60 ^e^ (6.53)	1.06 ^e^ (4.49)	1.52 ^bc^ (6.92)	0.70 ^e^ (7.01)	0.66 ^cd^ (6.81)
*S. auricolifolia*	14	1.51 ^e^ (17.78)	4.96 ^d^ (9.68)	1.43 ^bc^ (8.04)	1.33 ^f^ (10.27)	1.08 ^a^ (4.46)	0.77 ^f^ (7.64)	0.93 ^e^ (4.46)
*S. badaroi*	74	0.75 ^a^ (16.11)	3.69 ^a^ (7.35)	1.22 ^a^ (7.14)	0.78 ^b^ (10.04)	1.56 ^c^ (7.00)	0.69 ^de^ (5.80)	0.64 ^c^ (6.76)
*S. gazulensis*	23	0.72 ^a^ (14.61)	3.78 ^ab^ (9.12)	1.24 ^a^ (7.95)	0.73 ^a^ (7.18)	1.71 ^def^ (5.29)	0.63 ^b^ (5.67)	0.59 ^ab^ (5.40)
*S. hicesiae*	43	0.71 ^a^ (17.35)	3.87 ^b^ (9.38)	1.24 ^a^ (8.66)	0.73 ^a^ (9.65)	1.68 ^de^ (6.35)	0.60 ^a^ (7.30)	0.59 ^b^ (6.12)
*S. hifacensis*	128	1.00 ^b^ (17.60)	4.35 ^c^ (9.68)	1.46 ^bc^ (9.56)	0.87 ^c^ (8.79)	1.68 ^d^ (5.76)	0.66 ^c^ (5.08)	0.60 ^b^ (5.69)
*S. ichnusae*	20	0.96 ^b^ (16.82)	4.26 ^c^ (7.67)	1.39 ^b^ (9.49)	0.87 ^c^ (8.75)	1.60 ^c^ (8.44)	0.66 ^c^ (8.77)	0.63 ^c^ (8.19)
*S. mollissima*	20	1.04 ^b^ (11.35)	4.45 ^c^ (5.82)	1.51 ^cd^ (5.20)	0.87 ^c^ (7.20)	1.74 ^ef^ (4.59)	0.66 ^c^ (4.11)	0.58 ^a^ (4.73)
*S. oenotriae*	20	1.19 ^c^ (11.87)	4.93 ^de^ (6.26)	1.53 ^d^ (4.97)	0.99 ^d^ (8.09)	1.55 ^bc^ (7.40)	0.61 ^a^ (7.18)	0.65 ^cd^ (6.83)
*S. tomentosa*	20	1.03 ^b^ (11.23)	4.37 ^c^ (4.25)	1.39 ^b^ (5.05)	0.94 ^d^ (6.92)	1.49 ^b^ (4.22)	0.68 ^cd^ (5.06)	0.67 ^d^ (4.26)
*S. velutina*	39	1.23 ^c^ (13.57)	5.12 ^e^ (7.37)	1.66 ^f^ (6.15)	0.94 ^d^ (9.94)	1.78 ^f^ (9.90)	0.59 ^a^ (9.43)	0.57 ^a^ (9.60)

**Table 3:** Values of *J* index of the average silhouettes with lateral models LM1 to LM8. In bold, upper values obtained for each species.

Species	N	LM1	LM2	LM3	LM4	LM5	LM6	LM7	LM8
*S. andryalifolia*	24	**92.0**	91.8	88.3	91.9	91.9	84.2	88.5	89.0
*S. auricolifolia*	8	**92.9**	91.1	85.2	87.5	88.7	80.2	89.4	89.6
*S. badaroi*	70	91.0	91.0	90.4	**93.4**	91.7	86.3	89.3	90.3
*S. gazulensis*	32	90.6	88.4	90.4	90.8	**93.3**	87.7	91.4	90.0
*S. hicesiae*	48	88.1	87.3	89.2	**91.7**	90.1	88.7	87.5	86.8
*S. hifacensis*	128	91.9	91.0	90.4	**93.8**	92.0	86.3	89.1	90.5
*S. ichnusae*	20	**92.9**	91.7	88.3	90.5	91.9	82.9	89.4	91.8
*S. mollissima*	20	91.7	91.0	91.3	92.8	**93.4**	88.3	90.3	89.2
*S. oenotriae*	24	89.9	87.1	90.5	**92.0**	91.3	89.2	89.7	87.0
*S. tomentosa*	20	92.8	92.2	90.1	92.5	**93.2**	86.5	91.4	91.0
*S. velutina*	45	87.1	85.8	88.5	87.9	88.7	87.7	**89.1**	88.8

**Table 4:** Values of *J* index of the average silhouettes with dorsal models DM1, DM5 and DM6. In bold, upper values obtained for each species.

Species	N	DM1	DM5	DM6
*S. andryalifolia*	24	90.5	**93.7**	90.1
*S. auricolifolia*	8	**79.2**	**79.2**	68.0
*S. badaroi*	70	91.9	**94.2**	90.9
*S. gazulensis*	32	92.0	91.7	**93.1**
*S. hicesiae*	48	89.2	91.0	**91.4**
*S. hifacensis*	128	91.4	92.5	**93.4**
*S. ichnusae*	20	92.0	92.8	**92.9**
*S. mollissima*	20	91.4	90.9	**93.2**
*S. oenotriae*	24	91.8	**92.9**	85.7
*S. tomentosa*	20	92.2	**93.9**	91.5
*S. velutina*	45	89.8	87.8	**91.8**

**Table 5:** Results of Kruskal–Wallis and post hoc tests for the *J* index values (percent of similarity of the lateral views of seeds with models LM1, LM4 and LM5) of the 11 species of the *Silene mollissima* aggregate. Mean values and coefficient of variation (given in parentheses) are indicated for *J* index values with the different models. Values marked with the same superscript letter in each column correspond to populations that do not differ significantly at *p* < 0.05. N indicates the number of seeds analyzed. In bold, upper values obtained for each species.

Species	N	LM1	LM4	LM5
*S. andryalifolia*	24	89.2 ^c^ (1.64)	88.8 ^cd^ (1.68)	**89.9 ^b^** (1.72)
*S. auricolifolia*	8	**91.1 ^d^** (1.80)	85.2 ^ab^ (4.00)	85.8 ^a^ (2.61)
*S. badaroi*	70	89.8 ^cd^ (1.60)	**90.0 ^e^** (2.01)	89.7 ^b^ (1.42)
*S. gazulensis*	32	87.1 ^ab^ (2.80)	88.2 ^bcd^ (2.22)	**89.3 ^b^** (2.07)
*S. hicesiae*	48	86.1 ^a^ (3.18)	**88.1 ^bcd^** (2.76)	86.8 ^a^ (2.66)
*S. hifacensis*	128	90.2 ^cd^ (2.14)	**91.1 ^f^** (1.51)	90.1 ^b^ (1.45)
*S. ichnusae*	20	**89.3 ^cd^** (2.41)	88.2 ^bcd^ (2.70)	87.8 ^a^ (2.44)
*S. mollissima*	24	**87.7 ^b^** (2.07)	86.8 ^abc^ (3.27)	86.2 ^a^ (2.59)
*S. oenotriae*	24	86.2 ^a^ (3.83)	**87.8 ^bcd^** (2.25)	86.8 ^a^ (2.84)
*S. tomentosa*	20	**90.1 ^cd^** (1.40)	89.5 ^de^ (2.10)	89.7 ^b^ (1.69)
*S. velutina*	45	84.8 ^a^ (4.93)	84.8 ^a^ (4.58)	**85.8 ^a^** (3.61)

**Table 6:** Results of Kruskal–Wallis and post hoc tests for the *J* index values (percent of similarity of the dorsal views of seeds with models DM1, DM5 and DM6) of the 11 species of the *Silene mollissima* aggregate. Mean values and coefficient of variation (given in parentheses) are indicated for *J* index values with the different models. Values marked with the same superscript letter in each column correspond to populations that do not differ significantly at *p* < 0.05. N indicates the number of seeds analyzed. In bold, upper values obtained for each species.

Species	N	DM1	DM5	DM6
*S. andryalifolia*	24	87.3 ^cde^ (1.51)	**89.9 ^ef^** (1.43)	87.8 ^bcde^ (3.43)
*S. auricolifolia*	14	73.3 ^a^ (3.75)	73.7 ^a^ (3.49)	68.0 ^a^ (3.56)
*S. badaroi*	69	88.0 ^de^ (2.27)	**90.1 ^f^** (1.56)	88.4 ^bcde^ (2.78)
*S. gazulensis*	24	85.2 ^bcde^ (2.87)	85.4 ^bc^ (2.51)	**89.3 ^cde^** (1.54)
*S. hicesiae*	40	83.9 ^bc^ (2.77)	85.2 ^bc^ (2.98)	**88.8 ^bcde^** (1.64)
*S. hifacensis*	120	85.4 ^bcde^ (2.76)	86.3 ^bcd^ (2.67)	**90.4 ^de^** (1.42)
*S. ichnusae*	20	84.8 ^bcd^ (3.28)	86.6 ^cde^ (2.94)	**87.4 ^bc^** (3.31)
*S. mollissima*	20	84.4 ^bc^ (2.26)	84.6 ^bc^ (2.21)	**90.5 ^e^** (1.31)
*S. oenotriae*	20	86.3 ^bcde^ (2.19)	**88.7 ^def^** (1.77)	87.6 ^bcd^ (2.93)
*S. tomentosa*	20	**88.6 ^e^** (1.81)	**90.0 ^f^** (1.42)	86.1 ^b^ (2.31)
*S. velutina*	36	83.0 ^b^ (4.84)	83.2 ^b^ (5.04)	**87.8 ^bcde^** (3.04)

**Table 7:** Species and populations used in this work.

Species	Accession Code in BG-SAR	Locality (Date of Collection)	Seed Number (in Stock)	Mean Coordinates (WGS 84)	Mean Elevation (m a.s.l.)
*S. andryalifolia*	VM16/14	Canellas de Alabaida, Malaga, Spain (20 May 2014)	30	36°50′ N; 3°59′ W	500
*S. auricolifolia*	VM01/15	Santa Cruz, Orano, Algeria (12 May 2015)	16	34°42 N; 00°40′ W	316
*S. badaroi*	VM01/13	Capo Noli, Liguria, Italy (1 June 2013)	>200	44°11′ N; 8°25′ E	240
*S. badaroi*	VM06/13	Marciana, Isola d’Elba, Tuscan Archipelago, Italy (6 July 2012)	>200	42°48′ N; 10°08′ E	8
*S. badaroi*	VM07/13	Provence-Alpes-Côte d’Azur, Hyères, France (1 June 2013)	41	42°04′ N; 6°06′ E	10
*S. gazulensis*	VM17/14	Alcalá de los Gazules, Cadiz, Spain (15 June 2014)	48	36°27′ N; 5°43′ W	200
*S. hicesiae*	GM0313	Isole Eolie, Panarea, Sicily, Italy (2005)	66	38°38′ N; 15°03′ E	390
*S. hicesiae*	VM02/13	Isole Eolie, Panarea, Sicily, Italy (25 July 2013)	>200	38°38′ N; 15°03′ E	390
*S. hifacensis*	VM03/14	Passebret, Cap D’Or, Alicante, Spain (1 July 2013)	>200	38°40′ N; 00°08′ E	100
*S. hifacensis*	VM04/14	Morro de Toix, Calpe, Alicante, Spain (1 July 2013)	>200	38°37′ N; 00°01′ E	56
*S. hifacensis*	VM05/14	Cova Cendres, Cap D’Or, Alicante, Spain (1 July 2013)	>200	38°41′ N; 00°09′ E	80
*S. hifacensis*	VM06/14	Es Tossals, Cala Alabarca, Ibiza, Isole Baleari, Spain (16 June 2013)	92	39°03′ N; 01°22′ E	20
*S. hifacensis*	VM07/14	Illot de la Mona, Xabia, Alicante, Spain (1 July 2013)	49	38°48′ N; 00°11′ E	2
*S. hifacensis*	VM08/14	Morro de Toix, Calpe, Alicante, Spain (1 July 2014)	>200	38°37′ N; 00°01′ E	56
*S. ichnusae*	18/17	Capo Falcone, Stintino, Sardinia, Italy (1 July 2017)	67	40°58′ N; 08°12′ E	15
*S. mollissima*	VM10/14	Coma Freda, Maiorca, Isole Baleari, Spain (5 August 2014)	96	39°48′ N; 02°52′ E	650
*S. oenotriae*	VM04/13	Massiccio del Pollino, Basilicata, Italy (2 June 2013)	62	39°49′ N; 16°19′ E	350
*S. tomentosa*	VM03/13	Gibraltar Botanical Garden, Gibraltar (unknown)	64	36°07′ N; 05°20′ W	250
*S. velutina*	192/06	Cala del Morto, Isola di La Maddalena, Sardinia, Italy (15 July 2006)	>200	41°14′ N; 09°24′ E	3
*S. velutina*	54/17	Abbattoggia, Isola di La Maddalena, Sardinia, Italy (12 August 2017)	73	41°14′ N; 09°24′ E	3

## Data Availability

Not applicable.
